# First Detection of Bat Astroviruses (BtAstVs) among Bats in Poland: The Genetic BtAstVs Diversity Reveals Multiple Co-Infection of Bats with Different Strains

**DOI:** 10.3390/v13020158

**Published:** 2021-01-22

**Authors:** Anna Orłowska, Marcin Smreczak, Patrycja Potyrało, Arkadiusz Bomba, Paweł Trębas, Jerzy Rola

**Affiliations:** 1 Department of Virology, National Veterinary Research Institute, Al. Partyzantów 57, 24-100 Puławy, Poland; patrycja.potyralo@piwet.pulawy.pl (P.P.); p.trebas@piwet.pulawy.pl (P.T.); jrola@piwet.pulawy.pl (J.R.); 2 Department of Omics Analyses, National Veterinary Research Institute, Al. Partyzantów 57, 24-100 Puławy, Poland; arkadiusz.bomba@piwet.pulawy.pl

**Keywords:** astroviruses, bats, prevalence, Poland, phylogenetics

## Abstract

Background: Astroviruses (AstVs) are common pathogens of a wide range of animal hosts, including mammals and avians, causing gastrointestinal diseases, mainly gastroenteritis and diarrhea. They prompt a significant health problem in newborns and young children and economic losses in the poultry sector and mink farms. Recent studies revealed a growing number of bat species carrying astroviruses with a noticeable prevalence and diversity. Here, we demonstrate the first detection of bat astroviruses (BtAstVs) circulating in the population of insectivorous bats in the territory of Poland. Results: Genetically diverse BtAstVs (n = 18) were found with a varying degree of bat species specificity in five out of 15 bat species in Poland previously recognized as BtAstV hosts. Astroviral RNA was found in 12 out of 98 (12.2%, 95% CI 7.1–20.2) bat intestines, six bat kidneys (6.1%, 95% CI 2.8–12.7) and two bat livers (2.0%, 95% CI 0.4–7.1). Deep sequencing of the astroviral RNA-dependent RNA polymerase (RdRp) region revealed co-infections in five single bat individuals with highly distinct astrovirus strains. Conclusions: The detection of highly distinct bat astroviruses in Polish bats favors virus recombination and the generation of novel divergent AstVs and creates a potential risk of virus transmission to domestic animals and humans in the country. These findings provide a new insight into molecular epidemiology, prevalence of astroviruses in European bat populations and the risk of interspecies transmission to other animals including humans.

## 1. Introduction

Bats (Chiroptera) are unique mammals, with the ability to fly, that occur all over the world with approximately 1200 species [1]. They are a reservoir of many viruses which may cross the species barrier and infect humans and animals [2]. Multiple virological investigations worldwide revealed bat zoonotic pathogens such as lyssaviruses, Nipah and Hendra viruses, beta coronaviruses (SARS-like CoV, MERS-like CoV), filoviruses (Marburg and Ebola), hantaviruses, orthoreoviruses and astroviruses [2,3,4].

Astroviruses (AstVs) are common pathogens of animals and humans that cause gastrointestinal diseases, mainly gastroenteritis and diarrhea. They prompt a significant health problem in newborns and young children and economic losses in the poultry sector and mink farms. AstVs comprise a diverse group of small, non-enveloped single-stranded RNA viruses of positive polarity and 6.17 to 7.72 kb of genome size belonging to Astroviridae family. Two genera: avastroviruses, assigned to turkey, chicken and duck astroviruses, and mamastroviruses, including bat, ovine, porcine, bovine, domesticated animals, mink, rodents, marine mammals and novel human astroviruses are distinguished within the Astroviridae family [5,6]. Given the wide range of vertebrate hosts, the zoonotic spread to humans is possible from many mammals and, therefore, these viruses may pose a threat to public health.

Depending on the affected species, its immunological status and host age, astrovirus infections vary in clinical symptoms. Most astrovirus infections are assumed to be asymptomatic, but AstV infections can also be associated with diarrhea, hepatitis, nephritis and recently discovered encephalitis [5,7,8,9]. In bats, astroviruses were mostly found in apparently healthy animals without causing disease, similar to other viral infections, including highly pathogenic viruses, such as henipaviruses and filoviruses [10,11,12,13].

Bats are highly permissive to AstV infection. They may carry both host-restricted astroviruses as well as more diverse strains closely related to other members of the mamastrovirus genus, such as fox, murine, ovine, mink and human AstVs and even avian astroviruses of the avastrovirus genus. Phylogenetic analyses of the partial genome revealed a significant strain diversity of bat astroviruses forming numerous recognized and proposed species [6,14,15,16,17], supposedly generated both by intraspecies and interspecies recombination as well as interspecies transmission [18]. The high prevalence of diverse astrovirus strains from multiple hosts suggests that bats play a key role in astrovirus diversity and interspecies transmission [18].

Bat astroviruses were first detected in 2008 in Hong Kong and subsequently were detected in China, North America, Germany, Hungary, Czech Republic and Italy. They were detected in numerous bat species with the highest rate of occurrence in *Miniopterus magnater* bats (up to 100%), *Miniopterus schreibersii* (up to 80%), *Myotis daubentonii* (up to 64%) and *Myotis nattereri* (up to 40%). Astroviruses were detected in several bat species including Plecotus bats (*P. auritus*), Myotis bats (*M. bechsteinii*, *M. daubentonii*, *M. emarginatus*, *M. mystacinus*), Serotine bats (*Eptesicus serotinus*), Pipistrelle bats (*P. nathusii*, *P. pygmaeus*, *P. pipistrellus*), *Hypsugo savii*, *Nyctalus noctula*, *Vespertilio murinus* and *Rhinolophus hipposideros* [6,10,11].

Here, we demonstrate the first report on the detection of astroviruses in the population of different species of bats in Poland. Astroviral RNA was detected in different bat tissues, including the intestines, kidneys and bat livers. Phylogenetic analysis of Polish bat astroviruses (BtAstVs) based on partial region RNA-dependent RNA polymerase (RdRp) of astroviruses provides new data on the genetic diversity of AstVs crucial to the understanding of interspecies transmission and risk assessment to public health.

## 2. Materials and Methods

### 2.1. Samples

A total of 98 bats from the territory of Poland of both sexes, including individuals belonging to fifteen species: *Eptesicus nilsonii* (n = 2), *Eptesicus serotinus* (n = 22), *Nyctalus noctula* (n = 20), *Plecotus auritus* (n = 7), *Hypsugo savii* (n = 1), *Myotis dasycneme* (n = 2), *Vespertillo murinus* (n = 8), *Pipistrellus pipistrellus* (n = 4), *Myotis daubentonii* (n = 3), *P. pipistrellus/P. pygmaeus* (n = 4), *Myotis alcathoe* (n = 1), *Myotis mystacinus* (n = 2), *Myotis myotis* (n = 1), *Pipistrellus nathusii* (n = 3), *Myotis nattereri* (n = 1) and seventeen not identified bats, were collected in the frame of passive bat rabies surveillance and were tested for lyssavirus infection with negative results. All of the bat carcasses were kept frozen for several years before necropsy. Bat intestines, livers and kidneys were sampled during necropsy and were kept frozen at −20 °C in RNA later until RNA extraction. For the molecular identification of bats, fragments of wing membranes were taken. Where possible, during necropsy, sex was determined on the basis of estimation of the genitalia.

### 2.2. Molecular Classification of Bats Based on Genetic Markers

Molecular identification of bat species was performed based on cytochrome b sequence analyses as described before [19]. Total DNA was isolated from around 25 mm^2^ fragments of the wing samples using a DNA Mini Kit (Qiagen, Hilden, Germany) after overnight lysis using proteinase K. The lysate was loaded on DNA spin columns following the manufacturer’s instructions and the resulting DNA was used for amplification of the cytochrome b fragment as published before [19]. Amplicons were subjected to Sanger sequencing in both directions on the automated sequencer ABI PRISM 310 Genetic Analyzer (Applied Biosystem) using a BigDye Sequencing Kit (Applied Biosystem) with GeneScan Analysis Software and the same primers used for PCR. Bat classification was performed based on sequence mapping with reference sequences using the Basic Local Alignment Search Tool (BLAST) algorithm.

### 2.3. RNA Extraction

Suspension (20% *w*/*v*) was prepared from the intestines, livers and kidneys of bats in Minimum Essential Medium (MEM) (ATCC). Total RNA was extracted from 140 μL of homogenates using a QIAmp Viral RNA Mini Kit (Qiagen, Hilden, Germany) according to the manufacturer’s instructions. Nucleic acids were eluted in 50 μL elution buffer AVE and immediately subjected to AstVs detection using heminested RT-PCR or kept frozen at −80 °C for further investigation.

### 2.4. Molecular Detection of AstVs and Sequencing

The presence of AstVs RNA was investigated using Chu et al.’s [20] method. Reverse transcription was performed in a 20-μL volume mixture containing 50 ng of random hexamers and Superscript III Reverse Transcriptase (Life Technologies, Waltham, MA, USA) using the manufacturer’s recommendation. The most conserved region of RNA-dependent RNA polymerase (RdRp) gene was a target in heminested PCR for AstVs screening. Briefly, 1 μL of cDNA was added to a reaction mixture containing 0.1 mM dNTP, 2.5 mM MgCl_2_, polymerase DNA and primers mix of 10 μM each. In the next step, 1 μL of PCR product was used for heminested PCR. Amplification was performed in ProFlex thermocycler (Thermo Fischer Scientific, Waltham, MA, USA) with the following program: 1 cycle at 94 °C for 5 min followed by 40 cycles at 94 °C for 30 s, at 50 °C for 30 s, and at 68 °C for 1 min and a final elongation at 72 °C for 10 min. Amplicons were detected by separation in 1% agarose gel and after purification were sequenced in both directions on the automated sequencer ABI PRISM 310 Genetic Analyzer (Applied Biosystem, Waltham, MA, USA) using a BigDye Sequencing Kit (Applied Biosystem) with GeneScan Analysis Software.

### 2.5. NGS Sequencing

To increase the reliability of amplicons, deep sequencing was performed. At the beginning, the quantity and quality (A260/280 and A230/280) of DNA were measured with the use of a fluorimeter (Qubit 3.0, dsDNA HS Assay Kit, Thermo Fisher Scientific, Waltham, MA, USA) and spectrophotometer (NanodropOne, Thermo Fisher Scientific), respectively. In addition, the integrity of amplicons was checked by capillary electrophoresis (Fragment Analyzer, Agilent, Santa Clara, CA, USA) using DNF-488 High Sensitivity Genomic DNA Analysis Kit. Samples, which underwent quality control, were then normalized to equal concentrations. High-throughput sequencing (HTS) libraries were prepared from 1 ng of dsDNA, according to the Nextera XT (Illumina, San Diego, CA, USA) protocol. A dual indexing system (Illumina) was used for unique samples labelling. The libraries were then cleaned up with the use of magnetic beads—AMPure XP (Beckman Coulter, Brea, CA, USA). The quality and quantity of libraries were checked by Qubit (dsDNA BR Assay Kit, Thermo Fisher Scientific, USA) and Fragment Analyzer (NGS Fragment Kit DNF-473, Agilent, Santa Clara, CA, USA), respectively. Each library was normalized with the use of LN beads (Nextera XT DNA Library Prep Kit, Illumina), then pooled and diluted to a 20 pM concentration. Addition of 1% PhiX Control v3 (Illumina) was used as an internal control for sequencing. Pair-end sequencing (2 × 300 bp) was performed on MiSeq sequencer (Illumina) with the use of a V3 kit (Illumina).

### 2.6. Bioinformatics

Seqkit grep was used to find primers and reorient reads so that the amplicons sequences in the merged reads were in the same orientation. Quality control and adapter trimming was performed using the qiime cutadapt plugin. Further quality check and amplicon sequence variant detection was done by the qiime2 dada2 plugin. All obtained amplicon sequence variants (ASVs) with a length between 370 and 390 bp were extracted and visualized as an ASV relative frequency table.

### 2.7. Phylogenetic Analysis

Nucleotide sequences of 370 bp RdRp regions of Polish BtAstVs were aligned using Clustal W Multiple alignment 7.0.5.3. The similarity matrix was done using BLOSUM62 in BioEdit software v. 7.0.5.3. A phylogenetic tree was generated using a Maximum Likelihood tree with the appropriate evolutionary model and bootstrapped on the set of 500 replicates with the Mega X software [21]. To determine the phylogenetic relationship of astroviruses isolated in bat collected in Poland, 18 nucleotide sequences of AstVs were compared to reference sequences (available in GenBank database) guided by the closest relationships and geographic criteria.

## 3. Results

### 3.1. Prevalence of AstVs in Polish Bats

**Figure 1:**
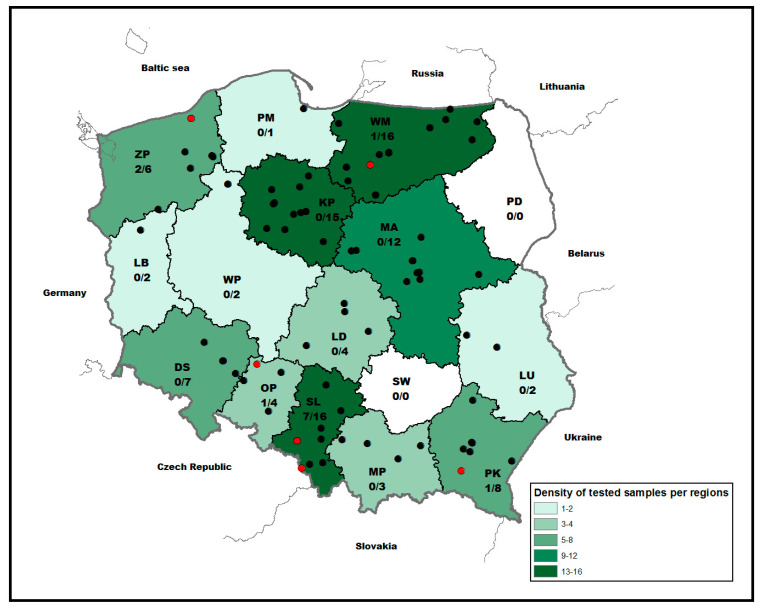
Map showing the voivodeships of Poland and the origin of bats included in the study and RT-PCR results (black: negative, red: positive). The number of positive to total samples per region is indicated. Abbreviation: MA (Masovia), DS (Lower Silesia), WP (Greater Poland), SL (Silesia), PM (Pomerania), LD (Łódź), MP (Lesser Poland), ZP (West Pomerania), LB (Lubusz), KP (Kuyavian–Pomeranian), OP (Opole), PD (Podlaskie), SW (Świętokrzyskie), WM (Warmian–Masurian), PK (Subcarpathian), LU (Lublin).

A total of 98 bat individuals originating from 14 out of 16 Polish voivodeships were tested for the presence of AstVs RNA (Figure 1). The largest number of samples originated from the Silesia (SL) followed by Warmian–Masurian (WM), Kuyavian–Pomeranian (KP) and Mazovian (MA) voivodeships (N = 12–16). Only a few samples were submitted from Lubusz (LB), Pomeranian (PM), Lublin (LU), Greater Poland (GP) and Lesser Poland (MP) voivodeships (Figure 1). While 37 (37.75.4%) and 49 (50.0%) were female and male, the sex of twelve bats (12.25%) could not be determined due to the decomposition stage of the carcasses and physical damages. Molecular classification based on cytochrome b genetic marker identified 81 bat specimens from fifteen different bat species. The vast majority of bats comprised *Eptesicus serotinus* (n = 22; 22.45%) followed by *Nyctalus noctula* (n = 20; 20.4%), *Vespertilio murinus* (n = 8; 8.16%) and *Plecotus auritus* (n = 7; 7.14%), whereas only a few other indigenous bat species were represented (Table 1). For 17 bats, genetic identification of the species failed.

Overall, 12 out of 98 (12.2%, 95% CI 7.1–20.2) bat intestines were positive for presence of BtAstVs RNA, whereas BtAstVs RNA was detected in six bat kidneys (6.1%, 95% CI 2.8–12.7) and two bat livers (2.0%, 95% CI 0.4–7.1). The percentage of positives was 75.0% (95% CI 46.8–91.1) in females and 25.0% (95% CI 8.9–53.2) in males. BtAstVs RNA was detected in five out of the 15 genetically identified bat species including *Nyctalus noctula* (n = 7), *Pipistrellus pipistrellus/Pipistrellus pygmaeus* (n = 2), *Plecotus auritus* (n = 1), *Myotis daubentonii* (n = 1) and *Vespertilio murinus* (n = 1). Table 1 summarizes the percentage of BtAstVs prevalence in different bat species included in the study.

Astroviral RNA was detected in bats collected from five out of 14 Polish voivodeships located in the northern (n = 2/5) and southern (n = 3/5) areas of the country (Figure 1). The majority of positive bats originated from Silesia (n = 7; 7.1% of all tested bats), which corresponded to the number of submitted bat carcasses (n = 16). The remaining positive bats were detected in the West Pomeranian (n = 2; 2%), Warmian–Masurian (n = 1; 1%), Subcarpathian (n = 1; 1%) and Opole (n = 1; 1%) regions. Temporal analysis revealed the highest number of AstVs found in bats collected in April (n = 6; 6.1%, 95% CI 2.8–12.7), September (n = 4; 4.1%, 95% CI 1.6–10.0), and October (n = 2; 2.0%, 95% CI 0.4–2.0).

### 3.2. Phylogenetic Resemblance of Polish BtAstVs

To perform the phylogenetic analysis, the 400-bp-long amplicons were submitted to Sanger sequencing that revealed the presence of a few AstVs in a single bat intestine sample. Co-infection of a single bat with several astroviruses was confirmed by deep sequencing in five out of 12 positive bat samples (41.7%) collected from three females and two males. No correlation between geographic region or particular season of collection was found for co-infected animals. Sequence homology between divergent astroviruses co-infecting a single bat ranged from 67.2 to 76.4%. Accession numbers of BtAstVs nucleotide sequences and % of raw data (reads) for individual viruses are shown in Table 2.

**Figure 2:**
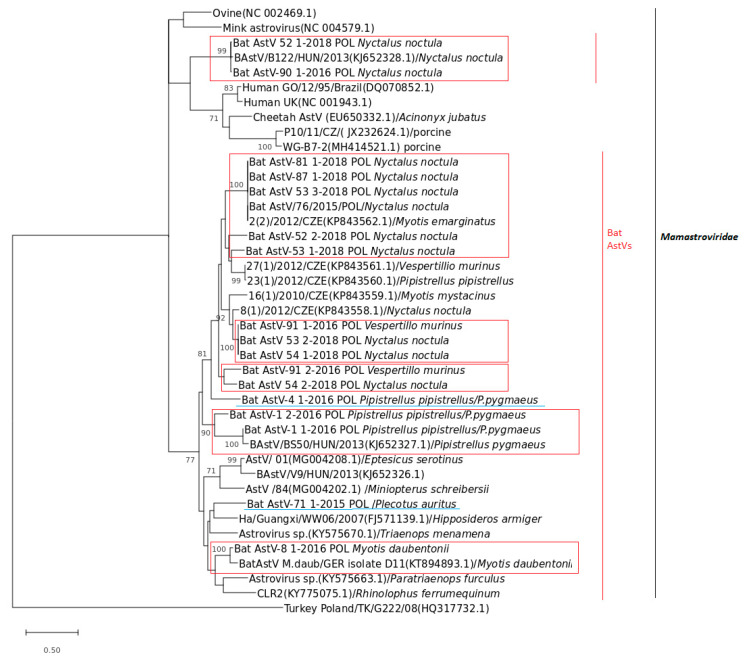
Phylogenetic tree based on the 382-bp-long nucleotide fragment of the RNA-dependent RNA polymerase (RdRp) gene of selected BtAstVs generated using the Maximum Likelihood tree with the appropriate evolutionary model with the Mega X Scheme 500. Bootstrap values over 70% from 500 replicates are shown next to the branches. Sequence Turkey_Poland/TK/G222/08 (HQ317732.1) was used as the outgroup. Polish BtAstV sequences determined in this study are in the red box in accordance to host restriction or underlined in blue.

Polish BtAstVs shared the common group of the Mamastroviridae family and were organized in a few sublineages suggesting a high diversity within all analyzed BtAstVs (Figure 2). The overall homology within Polish BtAstVs ranged from 54.9 to 100%. Phylogenetic analysis indicated that the majority of Polish BtAstVs clustered together with a wide group organized mostly with related European bat AstVs originating from the Czech Republic, Hungary and Germany. Two of the BtAstVs collected in noctule bats in 2016 (Bat Ast-90_1-2016_POL) and 2018 (Bat Ast-52_1-2018_POL) shared a common group with Hungarian BtAstV phylogenetically related to other mammalian AstVs isolated in porcine, human and cheetah (Figure 2). Some of the BtAstV sublineages were bat specific as BtAstVs circulating in Pipistrelle bats (*P. pipistrellus*/*P.pygmaeus*), *Nyctalus noctula* and BtAstVs circulating in *M. daubentonii*. The vast majority of Polish BtAstVs clustered with BtAstVs circulating mostly in noctule bats with single cases of infection in *V. murinus* and *M. emarginatus* in the Czech Republic and Hungary, respectively. Two Polish BtAstV isolates, Bat Ast-71_1-2015_POL and Bat AstV-4_1-2016_POL, collected from *P. auritus* and *P. pipistrellus*/*P.pygmaeus*, respectively, did not reveal bat species restriction and showed equal/comparable homology to all analyzed bat AstVs.

## 4. Discussion

In recent decades, a number of new viruses including zoonotic pathogens have been detected in bats as well as virus transmission from bats to human has been confirmed [1]. Bat–human disease transmission can involve intermediate hosts (domestic or wildlife animals) or spillover can happen directly, as was demonstrated for highly pathogenic viruses such as lyssaviruses and Nipah and Melaka viruses [16]. More than 200 new discovered viruses were associated with bats, and almost all were RNA viruses, probably owing to their great ability to adapt to challenging environmental conditions through a higher genetic variability [22,23]. The zoonotic potential of bat pathogens is increased by host traits such as long life spans, which allows for long-term chronic infection persistence, larger body size, long-distance migration, roosting characteristics of some bat species and resistance to viral infection [24].

The evidence of endemic AstV infections in Polish bats is shown for the first time in this paper. The only published data on AstVs epidemiology in Poland so far concerned the infections reported in avians [25,26] and children [27]. No studies of AstV prevalence in Polish mammals have ever been performed specifically in bats, and this is the first study showing for the first time the presence of AstVs infections in bats in Poland.

Based on evolutionary studies, bat astroviruses are known to have an exceedingly high genetic diversity affected both by interspecies transmission and recombination [18]. Numerous divergent strains that are yet to be classified and may reflect interspecies transmission events are detected. Therefore, more detailed sampling and characterization of animal astroviruses are still needed. Our data provide new knowledge on the spatial distribution of BtAstVs and bat species harboring AstVs. Phylogenetic analysis of Polish BtAstVs based on the RdRp region provides data crucial for studies on genetic diversity of AstVs in tracking host restriction and inter- or intraspecies transmission of AstVs.

The prevalence rate of astroviruses in samples collected from bats in Poland was 12.24%, and it was a similar rate to that in Italy (12.24%) but slightly higher than that observed in Hungary (6.94%) and lower than the overall prevalence of 25.8% previously reported in other European countries [10,16]. Astroviral RNA was detected in five out of 15 European bat species, previously recognized as bat AstV hosts, with the highest rate of prevalence in the Pipistrelle bat (50%, n = 2/4), *Nyctalus noctula* (42.1%, n = 6/19), *Myotis daubentonii* (33.3%, n = 1/3), followed by *Plecotus auritus* (14.28%, n = 1/7) and *Vespertilio murinus* (12.5%, n = 1/8). It concerned intestine samples, whereas, for kidneys, astroviral RNA was detected in five *Nyctalus noctula* bats and one *Myotis daubentonii* bat, but, for livers, only in two individuals of noctule bat. The lower prevalence rate of astroviruses in samples collected from bats in Poland compared to overall AstV prevalence in bats in Europe can be caused by the problem of bat conservation and the decay process of bat carcasses. Long-term storage of bats, particularly in non-frozen conditions, could lead to viral RNA degradation. Differences were found in the prevalence of AstV infection in females and males. Astroviral RNA was detected in bats collected from northern and southern Poland with high homology to German, Hungarian and Czech BtAstVs, respectively, that suggests an endemic circulation of astroviruses.

The majority of Polish BtAstVs revealed a high level of diversity (54.4–100% of nt sequence homology) and gathered phylogenetic groups formed by bat AstVs distinct from astroviruses circulating in other mammals, while two bat astroviral isolates collected from noctule bat in 2016 and 2018 were phylogenetically related to porcine, human and cheetah AstVs, indicating descent from the common ancestor. This also suggests the possibility of interspecies transmission of the virus both within a multispecies bat population and to other mammalian species including humans. Some bat sequences were previously recognized as highly permissive to infection with diverse astrovirus strains from multiple hosts as they clustered with strains from other species, including fox, cattle and mice [18]. Few Polish BtAstVs collected from Pipistrelle bats, *M. daubentonii* and three strains of BtAstVs isolated from noctule bat revealed host restriction and confirmed the bat species specificity of BtAstVs proposed by Fisher at al. [11]. Pipistrelle bats as well as *M. daubentonii* consist of one of the most common species distributed in Europe. They are sedentary species able to perform short- or medium-distance seasonal migrations (less than a few dozen) between summer and winter roosts. Individuals of *M. daubentonii* from Germany were reported to travel 260 km to the Miedzyrzecki Fortified Front in Poland and winter here. Mostly, they form separate breeding colonies but may share roosts with other bat species, i.e., the Pipistrelle bat shares roosts with *P. nathusii*, *E. serotinus* and *M. brandtii*, while *M. daubentonii* can share colonies with *N. noctula* or *N. leisleri*. The noctule bat overcomes long-distance seasonal migrations with the highest distance of 1600 km. Polish *N. noctula* hibernate in Hungary, Slovakia and Switzerland [28]. Forming large colonies, sharing common roosts during overwintering as well as long-distance migration from one roost to another contribute to intraspecies virus spread (host restricted) but also increase virus diversity and the risk of interspecies transmission particularly in the common roosts. A study in Germany revealed similar astrovirus sequences detected in bats from the same species in different habitats located at a distance of more than 600 km from one another. Simultaneously, predominant Polish astrovirus sequences (n = 10/18) revealed only little host restriction, and highly homologous sequences (homology up to 100%) were detected in *N. noctule* bats and a few individuals of *V. murinus* and *E. emarginatus*. Our results correspond to a recent study investigating frugivorous and insectivorous bats in Lao PDR and Cambodia, where a varying degree of host specificity in the detected astrovirus sequences was found [15]. Similar results of host restriction were found for other RNA viruses, i.e., alphacoronaviruses in Denmark [29]. The study revealed high host restriction and a close resemblance between the Danish *M.daubentonii* bat CoVs and British and German alphacoronaviruses even located roughly 330 km from each other, whereas other distinct Danish bat CoV sequences were present within each of the five bat species included in the study.

Deep sequencing and variant analysis performed for 400 bp region of RdRp revealed co-infection of five out of twelve positive bats with divergent BtAstVs (41.7% of total positive bats). Sequence identity matrix for BtAstVs infecting the single bat ranged between 67.2% and 76.4%. Previous papers demonstrated co-infection by diverse AstVs in turkey flocks [30] and pigs at the level of 13.9% [31]. While sampling bat colonies in China, Zhu et al. [12] detected several virus strains within one roost in a single cave at the same sampling day [12]. Another question is the astroviral RNA persistence in a bat individual. Due to the lack of virus isolates and experimental data, the pathogenicity and shedding pattern of astroviruses in bats is not yet well understood and more intensive investigations are needed. However, multiple sampling of tagged bat individuals over consecutive years in Germany revealed that most of the bats appeared to clear the virus. Those animals that did not clear harbored the virus for over a year, whereas the animals that were initially tested positive in RT-PCR were not positive again after a period of between 3 and 36 months [11].

A high level of co-infection provides frequent opportunity for recombination, especially between viruses of different lineages, and rapid generation of novel, divergent viruses of unknown pathogenicity. Numerous human recombinant strains, intraspecies recombinants have been reported so far. In 2010, a study conducted by Rivera et al. [32] suggested the possibility of interspecies recombination event between human and California sea lion astrovirus strain. A recombinant strain derived from porcine astrovirus and human HAstV-3 strains was reported from piglets and children of a different region of Colombia [33]. Different species of astroviruses that infect the same animal simultaneously promote the formation of new recombinants capable of infecting a new host and generate great diversity. The rate of generation of novel, divergent astroviruses is also affected by both intra- and interspecies recombination. The high biodiversity of the astroviral population can be affected by quasispecies evolution and the high ability of AstVs to generate genetic variability. A low replicative fidelity of the astroviral RNA polymerase, the kinetics of virus replication and undergoing recombination support a diversity of variant populations and the quick evolution of AstVs [34]. While these variants are generally less fit, they may quickly dominate and subsequently infect the new host if a sudden change in environment, such as immune pressure, shifts the corresponding fitness landscape [35].

The relatively high number of BtAstVs isolated in Polish noctule bats suggests *Nyctalus noctula* as a potential significant reservoir of astroviruses in the population of bats in Poland. However, six out of eight positive for BtAstVs noctule bats were collected at the same place and at the same sampling day at a park in the city center of Rybnik. Bats were found dead at the end of March, which would suggest that they woke up from hibernation and died due to the lack of food. In some individuals, hantavirus RNA was also detected (manuscript in preparation); therefore, we speculated that bats could die as a result of multiple or other viral infections.

Based on evolutionary analysis of astroviruses, an interspecies transmission pathway was strongly hypothesized as porcine strains were transmitted to cats and subsequently to humans, possibly involving other intermediary species [18]. Taking into consideration that 40 wintering *Nyctalus noctule* bats were found on the balcony on the second floor of a residential block in central Warsaw, the capital of Poland [36], as well as the fact that bats can come into close contact with both domestic animals and humans and contaminate the co-housed environment, the risk of transmission of astroviruses to human and other animals is possible. It is currently assumed that the risk posed by bats to the general public’s health is relatively low [3]; however, astrovirus transmission cannot be excluded. Bats are more frequently implicated in zoonotic virus emergencies [22]; therefore, pathogen surveillance in bats constitutes one of the most important preventive measures to public health.

Extra-intestinal diseases caused by AstVs were previously reported in avians including nephritis in chicken [8] and hepatitis in duck [9]. Here, we provide the evidence of AstVs RNA occurrence in kidney and liver bat samples. Bat astroviruses were previously detected in tissue samples including the brain, lung, intestine, heart and liver of Korean bats with a detection rate of 36.8% (21/57) [17]. A few cases of encephalitis caused by astrovirus infection were also reported in mink, bovines and humans [37,38,39]. However, in our study, all bat carcasses were submitted from regional laboratories post testing in the frame of passive bat rabies surveillance, and brains were extracted from carcasses. All bats were found dead and no information concerning the reason of death and the occurrence of clinical signs was available. However, it should also be underlined that numerous bat viral infections including astroviruses are asymptomatic, without causing disease and clinical signs [18].

In conclusion, our findings confirm that astroviruses circulate among the bat population in Poland, which creates a potential risk of virus transmission to domestic animals and humans in the country. A high rate of genetic diversity was found with a varying degree of bat species specificity among Polish BtAstVs. Deep sequencing of the astroviral RdRp region revealed co-infections of single bat individuals with highly distinct astrovirus strains which favor virus recombination and the generation of novel divergent AstVs. Therefore the significant diversity of astroviruses generated by their recombination and interspecies transmission should be continuously updated and under scientific control.

## Figures and Tables

**Table 1:** Prevalence of bat astroviruses (BtAstVs) in different bat species included in the study. n/a—not identified species of bats.

Species	No of Samples	No of Positives	Positives % (95% CI)
*Eptesicus serotinus*	22	0	0% (0.0–14.9)
*Nyctalus noctula*	20	7	35.0% (18.1–56.7)
*Plecotus auritus*	7	1	14.3% (0.07–51.3)
*Hypsugo savii*	1	0	0% (0–94.9)
*Myotis dasycneme*	2	0	0% (0–82.2)
*Vespertillo murinus*	8	1	12.5% (0.06–47.1)
*Pipistrellus pipistrellus*	4	0	0% (0.0–49.0)
*P.pipistrellus/pygmaeus*	4	2	50% (8.9–91.1)
*Myotis daubentonii*	3	1	33.3% (1.7–88.2)
*Myotis myotis*	1	0	0% (0–94.9)
*Pipistrellus nathusii*	3	0	0% (0–56.1)
*Myotis nattereri*	1	0	0% (0–94.9)
*Eptesicus nilsonii*	2	0	0% (0–82.2)
*Myotis alcathoe*	1	0	0% (0–94.9)
*Myotis mystacinus*	2	0	0% (0–82.2)
n/a	17	0	0% (0.0–18.4)

**Table 2:** BtAstVs involved in the study.

BtAstV	Collection Date	Host	Region	No. of Reads	Frequency- % of Total Reads	Accession Number
Bat AstV-1_1-2016_POL	2016	*P. pipistrellus/pygmaeus*—bat 1	ZP	41,139	64.9	MW399223
Bat AstV-1_2-2016_POL				17,094	26.98	MW399224
Bat AstV-4_1-2016_POL	2016	*P. pipistrellus/pygmaeus*—bat 4	ZP	53,166	86.11	MW399225
Bat AstV-8_1-2016_POL	2016	*M. daubentonii*—bat 8	WM	52,189	77.08	MW399226
Bat AstV-52_1-2018_POL	2018	*N. noctula*—bat 52	SL	27,479	43.5	MW399233
Bat AstV-52_2-2018_POL				4834	7.6	MW399234
Bat AstV-53_1-2018_POL	2018	*N. noctula*—bat 53	SL	7204	11.4	MW399235
Bat AstV-53_2-2018_POL				5044	7.18	MW399236
Bat AstV-53_3-2018_POL				4524	8.0	MW399237
Bat AstV-54_1-2018_POL	2018	*N. noctula*—bat 54	SL	10,576	31.48	MW399238
Bat AstV-54_2-2018_POL				4105	12.2	MW399239
Bat AstV-71_1-2015_POL	2015	*P. auritus*—bat 71	OP	55,241	88.7	MW399227
Bat AstV-76_1-2015_POL	2015	*N. noctula*—bat 76	SL	4793	30.15	MW399240
Bat AstV-81_1-2018_POL	2018	*N. noctula*—81	SL	29,338	89.56	MW399228
Bat AstV-87_1-2018_POL	2018	*N. noctula*—87	SL	10,726	57.3	MW399229
Bat AstV-90_1-2016_POL	2016	*N. noctula*—90	PK	22,590	91.57	MW399230
Bat AstV-91_1-2016_POL	2016	*V. murinus*—bat 91	SL	22,761	49.47	MW399231
Bat AstV-91_2-2016_POL				10,917	23.73	MW399232

Abbreviation: SL (Silesia), ZP (West Pomerania), OP (Opole), WM (Warmian–Masurian), PK (Subcarpathian).

## Data Availability

The collection of bats was performed in the frame of passive rabies surveillance in animals.
